# Controversies in the treatment of invasive urothelial carcinoma: a case report and review of the literature

**DOI:** 10.1186/s12894-015-0008-7

**Published:** 2015-03-13

**Authors:** Vicente Guillem, Miguel Angel Climent, Javier Cassinello, Emilio Esteban, Daniel Castellano, José Luis González-Larriba, Pablo Maroto, Carlos Camps

**Affiliations:** Instituto Valenciano de Oncología, Valencia, Spain; Hospital Universitario de Guadalajara, Guadalajara, Spain; Hospital Universitario Central de Asturias, Asturias, Spain; Hospital Universitario 12 de Octubre, Madrid, Spain; Hospital Clinico San Carlos, Madrid, Spain; Hospital Sant Pau, Barcelona, Spain; Consorcio Hospital General Universitario de Valencia and Universitat de Valencia, Valencia, Spain; Medical Oncology Department, Instituto Valenciano de Oncologia, C/Prof. Beltrán Báguena, 8-11 46009 Valencia, Spain

**Keywords:** Muscle-invasive bladder cancer, Urothelial carcinoma, Neoadjuvant chemotherapy, Adjuvant chemotherapy, Unfit

## Abstract

**Background:**

More than 429,000 patients worldwide are diagnosed with bladder cancer each year and muscle-invasive bladder cancer has an especially poor outcome. The median age at diagnosis is over 70 years, and many patients also have a substantial number of age-associated impairments that need to be considered when planning therapeutic interventions.

**Case presentation:**

Here, we report the case of a 63-year-old man with a cT3b urothelial carcinoma which was surgically removed. No neoadjuvant or adjuvant chemotherapy was administered. After 18 months a lung metastasis was confirmed and resected but no chemotherapy was given after surgery. Twelve months later, the patient relapsed and was treated with a combination of gemcitabine and cisplatin and after a decline in renal function the treatment was changed to a combination of carboplatin and gemcitabine which resulted in a partial response which lasted 8 months. Following this vinflunine was administered as a second line treatment.

Here we review the evidence available in the literature regarding the suitability of different treatment options for managing muscle-invasive bladder cancer at each step of the case presentation.

**Conclusion:**

Bladder cancer treatment requires a multidisciplinary approach. Although, depending on the clinical characteristics of the patient, there are some controversial points in the management of this pathology we hope that the scientific data and the clinical trials reviewed in this case report, can help to guide physicians to make more rational decisions regarding the management of these patients.

## Background

More than 429,000 patients worldwide are diagnosed with bladder cancer each year, which represents the fourth most common cancer in men [1]. The standard treatment for patients with muscle-invasive bladder cancer (MIBC) is radical cystectomy, although in a substantial proportion of these patients there is still a significant risk of distant recurrence after surgery [2]. Survival rates are poor for these patients: around 45% survive for five-years regardless of the type of treatment. In this context, neoadjuvant and adjuvant chemotherapy have been shown to significantly improve the outcome [3]. In metastatic settings there are front-line treatments that have a direct effect on survival which can be considered even in patients with impaired renal function.

Here we report the chronological evolution of a patient with a urothelial carcinoma with invasion into the muscular layer who was treated with radical cystectomy, and review and discuss the available treatment options during relapse and disease progression.

## Case presentation

A 63-year-old man was admitted at the emergency department of a general hospital after experiencing painless hematuria which was his only symptom; he had a history of smoking and hypertension. Laboratory tests showed results within normal limits: creatinine levels were 0.9 mg/dL and he had a glomerular filtration rate (GFR) greater than 60 mL/min. Urological ultrasonography revealed a 4 cm tumor on the right posterolateral wall of the bladder which appeared to be invasive.

Computed tomography (CT) of the abdominal and pelvic area showed a mass in the urinary bladder corresponding to stage 3 (cT3b) with apparent infiltration into the fat but without lymph node involvement. Cystoscopy and transurethral resection (TUR) of the tumor was carried out but residual tumor remained. Pathological examination confirmed a urothelial carcinoma with evidence of invasion into the muscular layer.

The patient did not receive neoadjuvant chemotherapy but surgical treatment was planned which consisted of radical cystectomy and Bricker-Wallace reconstruction. Histological examination of the resected specimen described a grade 3 urothelial carcinoma with infiltrations into the adjacent fat tissue. Twenty-two tumor-free lymph nodes were also removed, and the tumor was staged as a pT3bN0. The tumor margins as well as the urethers were microscopically negative and there was no in situ carcinoma. After surgery creatinine blood levels were 1.2 mg/mL and the GFR was higher than 60 mL/min.

The patient remained disease-free for 18 months until a single, newly-formed 1.6 cm lung nodule was discovered which was not accessible for biopsy using standard techniques. No mediastinal lymphadenopathies were found and there was no evidence of disease in any other location. At this point, the patient underwent resection of the mass and the histo-pathological examination revealed a 1.2 cm metastatic lesion from a urothelial carcinoma with tumor-free edges. No chemotherapy was administered after surgery.

Twelve months later, the patient developed multiple retroperitoneal and iliac lymph node relapses and a secondary II/IV hydronephrosis on the right side. Laboratory tests showed abnormal blood creatinine of 1.4 mg/dL and a GFR of 51 mL/min. He was treated with a combination of cisplatin and gemcitabine, but after two cycles his renal function worsened (serum creatinine was 1.6 mg/dL and his GFR was 45 mL/min). Therefore, the treatment was changed to a combination of carboplatin (AUC = 5) and gemcitabine. After 3 cycles of treatment the patient showed a very promising partial response, which was sustained for six cycles.

**Figure 1:**
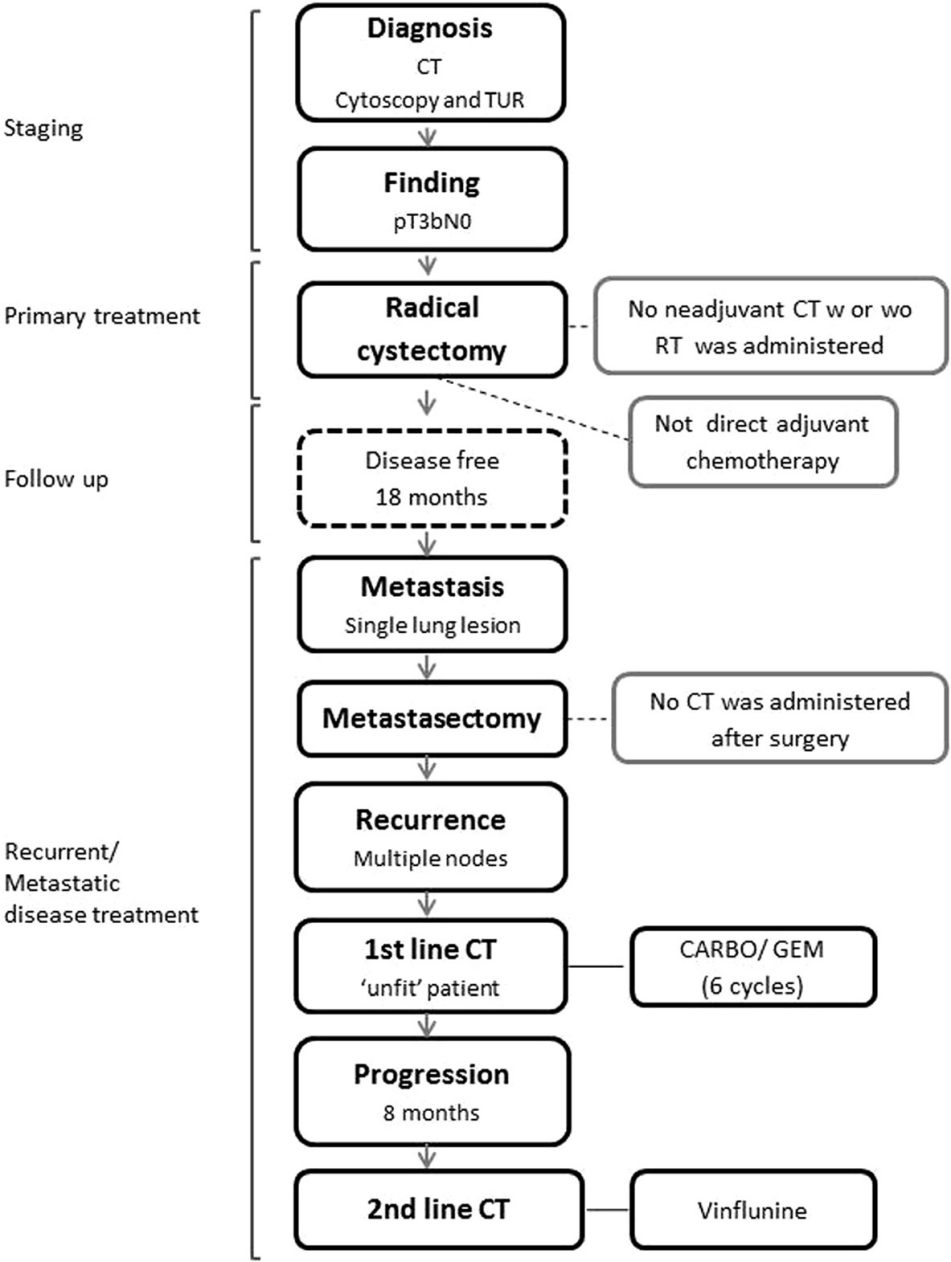
**Flow chart summarizing the management of the reported case.**

The patient remained progression-free for eight months, after which a lung and mediastinal lymph node relapse was found. He was treated with vinflunine at a dose of 320 mg/m^2^ and maintained good hematological function (See Figure 1 for a summary of patient’s management). The patient had remained stable for 4.5 months when progressive disease was detected. Appropriate palliative care was administered until he died 2 months later.

## Discussion

The standard treatment for patients with MIBC is radical cystectomy [4] as performed in the case reported here. Although this surgical approach leads to complete tumor excision in a substantial proportion of patients, individuals who have MIBC are at a significant risk of distant recurrence after surgery. Therefore, new therapeutic approaches like neoadjuvant chemotherapy, followed by radical surgery or adjuvant chemotherapy, after surgery have been explored in an attempt to reduce relapse rates.

In 2002, Sherif et al. reported the results of the Nordic Cystectomy Trial II, which compared the use of methotrexate/cisplatin as a neoadjuvant therapy prior to cystectomy compared to cystectomy alone, and found no statistically significant survival benefit with the neoadjuvant therapy after a five-year follow-up [5]. More recently, two relevant clinical trials have been reported: the S1011 trial by SWOG (the Southwest Oncology Group) which compared neoadjuvant chemotherapy consisting of M-VAC (methotrexate, vinblastine, adriamycin, and cisplatin) treatment combined with surgery versus surgery alone in 307 stage T2-4a patients [6], and the BA06 30894 trial which compared CMV (cisplatin, methotrexate, and vinblastine) combined with surgery or radiotherapy versus surgery or radiotherapy alone in 976 high-grade (T2-T4aN0-NXM0) bladder cancer patients [7]. The results from SWOG, showed that the median survival among patients assigned to cystectomy alone was 46 months [CI 95%: 25–60] compared to 77 months [CI 95%: 55–104] in the neoadjuvant group. Although the differences between the two groups did not reach statistical significance (*p* = 0.06), there was a trend that favoured the use of combination therapy. In the BA06 30894 study, neoadjuvant CMV treatment was associated with a 16% reduction in the risk of death [HR: 0.84, CI 95%: 0.72-0.99], corresponding to an absolute improvement of 6% in the 10-year overall survival (OS) [30% to 36%; *p* = 0.037].

In a systematic review that included 3,005 patients with advanced bladder cancer, which were randomly distributed among 11 randomized studies, the overall analysis did not favour the global use of neoadjuvant chemotherapy [HR: 0.89; CI 95%: 0.81-0.98; *p* = 0.22], although the subgroup receiving cisplatin-based neoadjuvant chemotherapy prior to cystectomy showed a 5% absolute increase in OS at 5 years follow-up [HR: 0.86; CI 95%: 0.77-0.95; p = 0.003] [8].

Furthermore, a retrospective study [9] and three meta-analyses [10-12] determined the OS and cancer-specific survival benefits in patients who receive cisplatin-based chemotherapy before radical cystectomy (level I evidence), and this should be considered the standard of care.

Based on all this evidence, and given that our patient was staged as a T3N0M0 and had a good performance status, neoadjuvant chemotherapy would be indicated in the form of 3–4 cycles of M-VAC or CMV.

Another controversial point in the management of this case (stage pT3bN0 MIBC) is the use of adjuvant chemotherapy, because the adoption of this therapeutic approach has been hampered by contradictory results. Cognetti et al. reported that there were no significant differences in OS and disease-free survival in patients with stage pT2G3, pT3-4, or N0-1 MIBC treated with four cycles of gemcitabine and cisplatin (GC) compared to no adjuvant chemotherapy after radical cystectomy [HR 1.29, p = 0.24 for OS] [13]. In contrast, a randomized phase III trial comparing four cycles of PGC (paclitaxel, gemcitabine, and cisplatin) with no adjuvant treatment in 142 patients with stage pT3-4 and/or pN+ found that OS was significantly improved in the PGC arm [five-years OS: 60% vs 31%, *p* < 0.0009] [14].

According to the clinical evidence, the current data point to a reduced risk of recurrence in patients with stage pT3-pT4a and/or pN+, M0 when treated in an adjuvant setting. Recently, a meta-analysis with a total of 945 patients included in nine randomized clinical trials provided further evidence of a benefit in terms of OS [HR: 0.77, *p* = 0.049] in patients receiving adjuvant cisplatin-based chemotherapy after surgery [15].

Taking all this data together, it seems that the optimum combination of chemotherapeutic agents for adjuvant regimens should be based on combining cisplatin with at least two other drugs. Therefore, the latest edition of the National Comprehensive Cancer Network (NCCN) guidelines recommend the use of a combined chemotherapy scheme such as M-VAC or GC (for at least three cycles) in patients with stage pT3-pT4a and/or N+ and M0 bladder cancer as well as for patients with stages pT2 or earlier, or cancers without nodal or vascular invasion whose tumors express high levels of p53 (higher than 20%) [2] (Table 1.A).

**Table 1:** **Summary of the current chemotherapeutic options in muscle invasive bladder carcinoma**

**Treatment MIBC**		**Schemes**	**References**
**A. cT3, cT4a** (negative nodes)			
Neoadjuvant CT		○ MVAC	6, 7, 8, 9
		○ HD-MAV	
Adjuvant CT		○ GC	2, 13*, 14, 15
		○ PGC	
**B. Metastatic disease**			
1st line	‘Fit’ for Cisplatin (GFR ≥ 60 mL/min; PS 0–1)	○ GC	21, 22, 23
		○ MVAC	
		○ PGC	
	‘Unfit’ for Cisplatin (GFR < 60 mL/ min; PS 2)	○ Combinations based in Carboplatin (CaG/M-CaV)	24, 25, 26, 27
		○ Monotherapy (P, G, Vinflunine)	
2nd line	‘Fit’	○ Vinflunine	35
	‘Unfit’	○ Clinical trial	

(*) No significant differences reported; A: Adriamycin, C: cisplatin, Ca: carboplatin, G: gemcitabine, M: methotrexate, P: paclitaxel, V: vinblastine, CT: chemotherapy.

Returning to our patient, after radical surgery he relapsed with a single metastatic lung lesion. The issue to discuss here is if the metastasis should have been resected and if subsequent chemotherapeutic treatment should have been administered.

Several reports have shown that, metastasectomy increases the number of long-term survivors in urothelial cancer [16,17] and provides the opportunity for an accurate histological diagnosis. This point is important due to the high incidence of secondary neoplasms in patients with bladder cancer [18]. The study from Kanzaki and colleagues showed that when assessing the need for resection, the number of metastases should be taken into account because the five-year OS rate of patients with a single metastasis was 85.7%, while that of patients with multiple metastases was 20.0% (*p* = 0.009) [19]. A retrospective survey of 44 patients (between 1991 and 2008) with distant metastasis of bladder or upper urinary tract cancers that underwent complete resection of all detectable metastases indicated that the criteria for metastasectomy were the following: absence of contraindication for surgery, no mediastinal nodal involvement, absence of extrapulmonary disease, and a number of lesions less than five [20]. Therefore, in our patient, the decision to surgically resect the metastasis was clearly justified.

Regarding the subsequent use of chemotherapy, considering the short period until the diagnosis of the metastasis, and the fact that no adjuvant or neoadjuvant chemotherapy was given, implementation of adjuvant chemotherapy is a reasonable recommendation.

Twelve months after metastasectomy, our patient progressed. At this point, it we considered administrating a cisplatin-based regimen, which directly benefits survival. However, over 50% of patients with urothelial cancer are ‘unfit’ (a GFR less than 60 mL/min and/or a performance status of two or more) or present comorbidities (cardiovascular, neuropathy, or hearing loss) and therefore are not eligible for cisplatin-based regimens [19].

Thus, for ‘fit’ patients, regimens with cisplatin (GC, M-VAC, or PGC) are valid treatment options [21-23], but GC remains the combination with the best ratio of efficacy/tolerability. In ‘unfit’ patients, regimens without cisplatin are used, frequently in monotherapy with either gemcitabine, carboplatin, vinflunine, or a taxane [24]. In an EORTC 30986 study on ‘unfit’ patients, treatment with carboplatin/ gemcitabine and methotrexate/carboplatin/vinblastine (M-CAVI) showed no significant differences in efficacy between the two treatment groups, and a higher incidence of severe acute toxicities for those patients receiving M-CAVI [25]. A review of the most recent clinical trials regarding this issue pointed to gemcitabine with carboplatin as a reasonable first-line regimen for ‘unfit’ patients [26]. It is also possible to use vinflunine in these patients, which in this study gave an overall response rate of 14.6% [CI 95%: 9.4-21.2], a remarkable 13% [CI 95%: 6- 24%] in patients with renal failure, and 21% [CI 95%: 13-32%] in patients 65 years or older [27].

Eight months after receiving a combination of platinum agents and gemcitabine as a first-line treatment, our patient relapsed. In this context, small phase II trials with paclitaxel, docetaxel, oxaliplatin, ifosfamide, topotecan, lapatinib, gefitinib, bortezomib, and pemetrexed achieved response rates of less than 10% [28-31]. Paclitaxel and gemcitabine have obtained some good results in some studies, depending on the response to previous treatments, but there are no comparative studies confirming the value of this combination as a second-line treatment [32]. In 2011, three adverse risk factors: performance status (PS), hemoglobin (Hb) level, and liver metastasis, that predict OS were validated, allowing patients with platinum-refractory disease on second-line chemotherapy to be classified into four risk groups with different outcome expectations [33]. In a retrospective analysis, Sonpavde et al., found that PS > 0, Hb level < 10 g/dl, liver metastasis, and a shorter time period between prior and new chemotherapy treatments were independent significant prognostic factors for OS and PFS in the setting of second-line therapy for advanced urothelial carcinoma [34].

Currently, the only drug approved by regulatory agencies (EMA) and recommended by scientific guidelines (ESMO, UAE, SEOM, etc.) is vinflunine, which, as a second-line treatment, achieves a response rate of 15%, and disease control rate of 67%, with acceptable toxicity rates [27]. One randomized phase III trial compared the use of vinflunine and best supportive care (BSC) versus BSC alone in the treatment of patients who progressed after a first-line platinum-containing regimen, and the median OS was significantly longer in the group treated with vinflunine [6.9 vs. 4.3 months, *p* = 0.040]. The overall response rate, disease control rate, and PFS were all statistically significant and favored vinflunine (*p* = 0.006, *p* = 0.002, and *p* = 0.001, respectively), while still maintaining a good quality of life, with limited toxicity [35]. Therefore, in our patient and in cases of advanced or metastatic urothelial cancers who are considered ‘unfit’ for chemotherapy as a second-line treatment, there is scientific evidence supporting the use of vinflunine as a reasonable therapeutic option (Table 1B).

## Conclusion

Patients with MIBC should be treated within the framework of a multidisciplinary team. Before resection, the use of neoadjuvant chemo/radiotherapy should be considered when clinically indicated, and is not associated with an increased risk of postoperative morbidity and mortality; it is important that renal function be monitored in these types of patients. Our case report and the data reviewed here demonstrate that in the context of advanced and metastatic cancer there are several chemotherapeutic options that improve survival, even in the case of ‘unfit’ patients. Vinflunine has been demonstrated to increase OS in patients after treatment with a platinum-containing regimen, and at present is the only drug approved in the second-line treatment setting.

## Consent

Written informed consent was obtained from the patient’s next of kin for publication of this case report and any accompanying images. A copy of the written consent is available for review by the Editor of this journal. Authors also obtained approval from the Instituto Valenciano de Oncología’s Ethics Review Board for the publication of this report.
